# Changes in rays’ swimming stability due to the phase difference between left and right pectoral fin movements

**DOI:** 10.1038/s41598-022-05317-5

**Published:** 2022-02-11

**Authors:** Hiroaki Sumikawa, Yoshikazu Naraoka, Takashi Fukue, Tasuku Miyoshi

**Affiliations:** 1 Mechanical and Aerospace Engineering, Division of Science and Engineering, Iwate University, 4-3-5 Ueda, Morioka, Iwate 020-8551 Japan; 2 Mechanical Engineering, Division of Engineering, Kanazawa Institute of Technology, 3-1 Yatsukaho, Hakusan, Isikawa 924-0838 Japan

**Keywords:** Behavioural ecology, Fluid dynamics

## Abstract

Swimming motions of rays that swim using undulation locomotion are not always symmetrical; there may be a phase difference between the left and right pectoral fins. However, few studies on the swimming of rays have mentioned left and right pectoral fin movements. Moreover, the effects of movements of the left and right pectoral fins on swimming have not been clarified. This paper describes a computational study of phase differences of pectoral fin movements in the swimming of rays with the validity of fluid analysis methods. The movement and shape of the ray were made based on previous biological research and pictures. An overset grid was used to reproduce the ray’s complex motions. The analysis was performed under four phase difference conditions: 0 $T$ ($T$ is the period), 0.25 $T$, 0.5 $T$, and 0.75 $T$. The results show that a phase difference between the left and right pectoral fin movements affects swimming stability and maneuverability but not propulsive efficiency. We suggest that the phase difference in pectoral fin movements is essential for the swimming of rays, and rays adjust the phase difference between the movement of the left and right pectoral fins to suit their purpose.

## Introduction

Rays use a unique swimming method called the median and paired fin (MPF) mode, while most fishes use the body and/or caudal fin (BCF) mode^1^. The ray's motion is divided into two movements: (1) mobuliform oscillation, underwater flapping flight, and (2) rajiform undulation, pectoral fins undulate from anterior to posterior along with pectoral fins^2^. Studies on both swimming modes of rays have traditionally been conducted to understand the ecology of rays and apply engineering applications.

Previous studies have clarified the swimming methods and mechanisms of undulate rays. The freshwater stingray *Potamotrygon orbignyi*'s swimming behavior was investigated and revealed that a leading-edge vortex highly affects swimming efficiency^3^. The relationship between swimming speed and the motion of skates was investigated, and it was found that the swimming speed increased as the amplitude and wave number increased^4^. In a simple model analysis, the ratio of the two methods of thrust generation (added mass and circulation) was examined, and it was found that the ratio of thrust generation by circulation was higher^5^. The relationship between the aspect ratio of the pectoral fin and swimming performance was investigated, and it was found that thrust and propulsive efficiency increased as the aspect ratio of the pectoral fin increased^6^. It was also clarified that the propulsive efficiency is improved by using the ground effect in both oscillation and undulation^7–10^. In addition, it has been shown that motions vary depending on the species and the depth at which they live^11^.

In KAIYUKAN, which is one of the biggest aquaria in Japan, pitted stingray *Dasyatis matsubarai* and sharpnose stingray *Dasyatis acutirostra* swim with a phase difference between the left and right pectoral fins movements (Supplement Movies 1, 2). The swimming motion of *D. matsubarai* and *D. acutirostra* undulates their pectoral fins from front to back (rajiform). It has been suggested that the different movements of the left and right pectoral fins affect turning and maneuverability in swimming rays. Shi et al. examined the effects of using different motions for the left and right pectoral fins and reversing the direction of wave propagation on swimming and showed that they improved turning ability^12^. In addition, Yang et al. showed that it could change direction by reversing the propagation direction of the left and right pectoral fin movements or making the amplitude of the left and right pectoral fin movements different^13^. Finally, in experiments with a stingray robot, Li et al. showed that travel direction could be controlled by changing the speed at which the left and right pectoral fins moved^14^. However, in this study, phase differences between the pectoral fins’ movements are often observed even when swimming straight ahead (Supplement Movies 1, 2). To the best of our knowledge, there is no study on the phase difference of movements of the left and right pectoral fins on straight-line swimming. However, previous studies have shown that differences in the movement of the left and right pectoral fins affect rays' turning ability and maneuverability^12–14^. Therefore, we hypothesized that the phase difference in the movement of the left and right pectoral fins during straight-line swimming would increase swimming stability and maneuverability. Since there is a trade-off between stability and maneuverability, it is not easy to have features of both. However, fishes that can do both will have a better chance of survival. The pufferfish can swim with maneuverability and stability by the vortices generated by its body and the way it moves its fins^15–17^. In insects and rajiform and mobuliform rays, stability has been shown to be affected by changes in the amplitude and speed of the left and right pectoral fin movements and the bending angle of the pectoral fins^13,14,18–20^. Although these previous studies were based on the assumption of turning, the amplitude and speed difference of the left and right pectoral fin movements are expected to maintain posture effectively even during straight-line swimming. However, it still is not clear whether the phase difference increased or decreased swimming stability and maneuverability. Investigating the effect of the phase difference between the left and right pectoral fin movements on swimming performance is essential to understanding the biology and ecology of *D. matsubarai*, *D. acutirostra,* and other stingrays that have similar motions, because they frequently perform phase-difference swimming. By increasing the maneuverability and stability of swimming, the fish may benefit in the following ways. The fish can take the shortest route from point A to point B by improving swimming stability, thus reducing energy consumption^21^. In addition, since vision blurring is reduced by improved swimming stability, the target's movement (enemy or prey) can be accurately understood. Improved swimming maneuverability will make it easier for fish to catch their prey and evade enemies^22^. It is also helpful for improving the swimming performance of stingray-type underwater robots. Those robots are better than propellers in waters littered with seaweed and plastic debris or in waters with high hydrostatic pressure^23,24^.

This study aims to elucidate the effect of the phase difference between pectoral fin movements on swimming. Computational Fluid Dynamics (CFD) analysis was used instead of actual fish observation to give precise phase differences to the left and right pectoral fin movements. We calculated the forces acting on the ray's body and investigated the phase difference between the left and right pectoral fin movements on propulsive efficiency and swimming stability. Our results showed that the phase difference between both pectoral fins movements affects swimming stability but not propulsive efficiency. The contributions and implications of this study are elucidations of (1) what is the effect of the phase difference between movements of the left and right pectoral fins on undulation propulsion and (2) why do stingrays swim straight ahead by phasing their left and right pectoral fin movements.

## Methods

### Analytical targets

**Figure 1:**
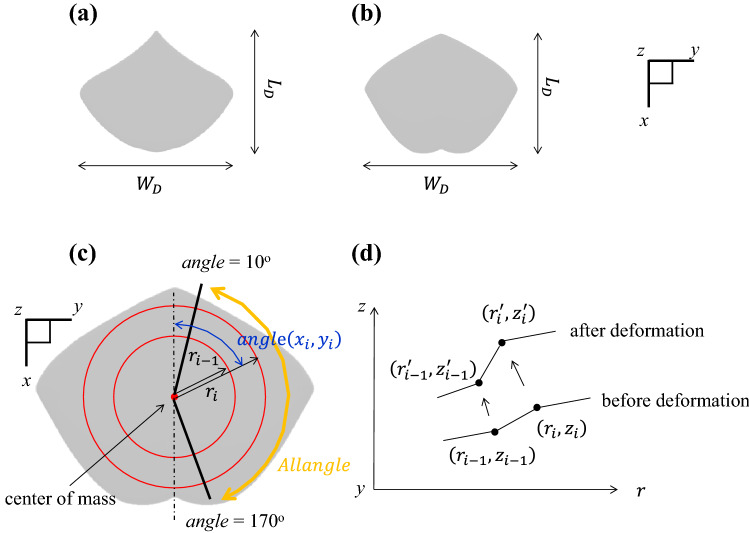
Analytical targets and description of motion. (**a**) Analytical model of *D. acutirostra.* (**b**) Analytical model of *D. matsubarai.* (**c**) Description of motion, (**d**) the relationship between any two points on the surface before and after the deformation.

Two species of undulation motion rays with different pectoral fin shapes bred in KAIYUKAN were analyzed: sharpnose stingray *Dasyatis acutirostra* and pitted stingray *Dasyatis matsubarai* (Fig. 1a,b). Blender 2.79^25^ was used to construct stingray models from pictures^26,27^ as accurately as possible; Blender is a free and open-source 3D creation suite used to make realistic characters for movies, etc. Detailed information on how to construct models using Blender is provided in our previous paper^28^. To focus on the effects of pectoral fin movements, we did not consider the body's shape as in the previous studies^12,29^. The height and disk width (*W*_*D*_) of all models were set to 0.01 m and 0.44 m, respectively, considering the previous studies^30,31^. The disk length of each model was determined from *W*_*D*_, referring to the aspect ratio of the rays' photographs^26,27^; the disk length (*L*_*D*_) of *D. acutirostra* and *D. matsubarai* are 0.348 m and 0.344 m, respectively.

### Motion

The motion was given to satisfy the following equations:

1 $$z = \left\{ {\begin{array}{*{20}l} {A\;\sin \left( {\omega \left( {t - kT\left( {\frac{{angl{\text{e}}\left( {x_{i} ,y_{i} } \right) - 10^{{\text{o}}} }}{{All\;angl{\text{e}}}} - \theta } \right)} \right)h_{1} h_{2} } \right.} \hfill & {\left( {10^{{\text{o}}} \le angl{\text{e}}\left( {x_{i} ,y_{i} } \right) \le 170^{{\text{o}}} } \right)} \hfill \\ {A\;\sin \left( {\omega \left( {t - kT\left( {\frac{{350^{{\text{o}}} - \left( {angl{\text{e}}\left( {x_{i} ,y_{i} } \right) - 10^{{\text{o}}} } \right)}}{{All\;angl{\text{e}}}}} \right)} \right)h_{1} h_{2} } \right.} \hfill & {\left( {190^{{\text{o}}} \le angl{\text{e}}\left( {x_{i} ,y_{i} } \right) \le 350^{{\text{o}}} } \right)} \hfill \\ \end{array} } \right.$$

2 $$\begin{array}{c}{h}_{1}=a{r}_{i}^{3}+b{r}_{i}^{2}+c{r}_{i}\end{array}$$

3 $$h_{2} = \left\{ {\begin{array}{*{20}l} {d\left( {angle\left( {x_{i} ,y_{i} } \right) - 10^{ \circ } } \right)^{2} + e\left( {angle\left( {x_{i} ,y_{i} } \right) - 10^{ \circ } } \right)} \hfill & {\left( {10^{ \circ } \le angle\left( {x_{i} ,y_{i} } \right) \le 170^{ \circ } } \right)} \hfill \\ {d\left( {350^{ \circ } - \left( {angle\left( {x_{i} ,y_{i} } \right) - 10^{ \circ } } \right)} \right)^{2} + e\left( {350^{ \circ } - \left( {angle\left( {x_{i} ,y_{i} } \right) - 10^{ \circ } } \right)} \right)} \hfill & {\left( {190^{ \circ } \le angle\left( {x_{i} ,y_{i} } \right) \le 350^{ \circ } } \right)} \hfill \\ \end{array} } \right.$$

4 $$\begin{array}{c}{\left({r}_{i}-{r}_{i-1}\right)}^{2}+{\left({z}_{i}-{z}_{i-1}\right)}^{2}={\left({r}_{i}^{\mathrm{^{\prime}}}-{r}_{i-1}^{\mathrm{^{\prime}}}\right)}^{2}+{\left({z}_{i}^{\mathrm{^{\prime}}}-{z}_{i-1}^{\mathrm{^{\prime}}}\right)}^{2}\end{array}$$

5 $$\begin{array}{c}angle\left({x}_{i},{y}_{i}\right)= angle\left({x}_{i}^{^{\prime}},{y}_{i}^{^{\prime}}\right).\end{array}$$

Equation (1) represents the amount of movement of the model surface in the *z*-axis direction, where $A$ is the amplitude of the pectoral fin tip, $\omega$ is the angular velocity, $t$ is time, $k$ is the wavenumber, $T$ is the period, *angle*(${x}_{i},{y}_{i}$) is the angle made by the line connecting the center of rotation and any point (${x}_{i},{y}_{i}$) on the model surface with the *x*-axis, *Allangle* is the range where the motion is given (160°), and $\theta$ is the phase difference between the movements of the right and left pectoral fins (Fig. 1c). ${h}_{1}$ is the weighting from the center to the radial direction: it is necessary to set the amplitude at the ray's center to zero and increase the amplitude toward the pectoral fin tip (*a* = 119.786, *b* = -7.957, *c* = 0.498). ${h}_{2}$ is the weighting in the circumferential direction: it is necessary to increase the amplitude from the anterior to the tip of the pectoral fin and decrease the amplitude from the tip of the pectoral fin to the posterior (*d* = − 1.563 × 10^–4^, *e* = 0.025). Equation (4) is the condition in which the distance between two neighboring points in the same radial direction is equal before and after the movement (Fig. 1d). $r$ is the distance between the center of rotation and any point (${x}_{i},{y}_{i}$), defined as $\sqrt{{x}_{i}^{2}+{y}_{i}^{2}}$. Equation (5) defines *angle*(${x}_{i},{y}_{i}$) as being constant before and after the move (Fig. 1c). The variables after the move are marked with '. Variables used in the analysis are *A* = 0.089 m, *T *= 0.499, *k *= 1.270, and *ω *= 12.599 rad/s. Videos of the created motion from the front and the side are shown in the “Supplement” (Supplement Movies 3, 4).

### Analytical conditions

Analysis cases were conducted with eight conditions: two types of pectoral fin shape (Fig. 1a,b) and four types of phase difference (0 $T$, 0.25 $T$, 0.5 $T$, and 0.75 $T$). These conditions were set for investigating the effects of phase differences between left and right pectoral fin movements on swimming and how these effects vary with pectoral fin shape.

### Numerical methods

**Figure 2:**
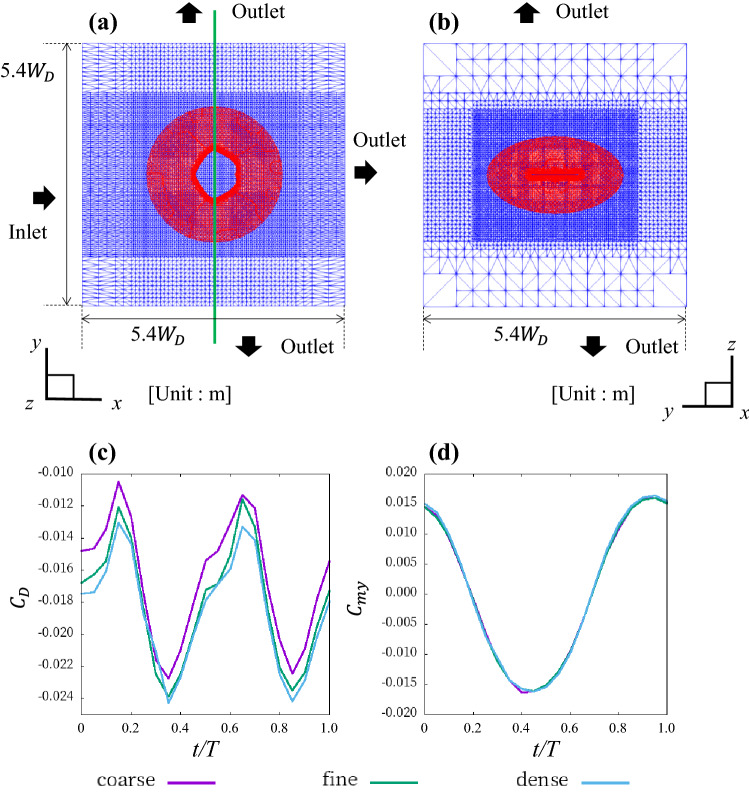
Meshes for CFD simulation and differences in force between different meshes. (**a**) Meshes at the coronal plane of the whole fluid region. (**b**) Frontal cross-section of the fluid region at the green line in (**a**). The red region is the overset region. (**c**,**d**) Comparison of the instantaneous drag coefficient and the moment coefficient around the *y*-axis of *D. matsubarai* between the coarse, fine, and dense mesh.

A CFD simulation of the ray models in the water flow was performed using OPENFOAM, an open-source finite volume method CFD toolbox^32^, to calculate the forces acting on the rays in each axial direction and the moment around each axis*.* The governing equations were the continuity equation and the three-dimensional incompressible Reynolds-averaged Navier–Stokes equation, expressed by:

6 $$\begin{array}{*{20}c} {\nabla \cdot u = 0} \\ \end{array}$$

7 $$\begin{array}{*{20}c} {\frac{\partial u}{{\partial {\text{t}}}} + \nabla \cdot \left( {uu} \right) = - \nabla p + \nabla \cdot \left( {v\nabla u} \right) + \nabla \cdot \left[ {\nu \left\{ {\left( {\nabla u} \right)^{T} - \frac{1}{3}\nabla \cdot uI} \right\}} \right], } \\ \end{array}$$

where $u$ is the velocity vector,* t* is the time, *p* is the static pressure divided by the reference density, $\nu$ is the kinematic viscosity, and *I* is the unit tensor. The Reynolds number was defined regarding the previous studies^3^ as:

8 $$\begin{array}{c}{R}_{e}=\frac{U{L}_{D}}{\nu },\end{array}$$

where $U$ (/ms) is the given flow speed^3^ (1.5 × *L*_*D*_/ms), ${L}_{D}$ (m) is the length of the ray models*,* and $\nu$ is the kinematic viscosity of water at 20 °C (1.0 × 10^–6^ m^2^/s). The Reynolds number in this study is 1.8 × 10^5^; considering this, we used the *k*–*ω* shear stress turbulence model^33,34^. The *k*–*ω* shear stress turbulence model is a type of Reynolds-averaged Navier–Stokes equation (RANS) turbulence model that is widely used to calculate for the fish swimming flow^35–37^. The overset grid method was used in this study; it is a generic implementation of overset meshes. For both static and dynamic cases, cell-to-cell mapping between multiple, disconnected mesh regions is employed to generate a composite domain^38,39^. This method permits complex mesh motions and interactions without the penalties associated with deforming meshes. The process is described in detail by Noack^40^. The calculation volume was 5.4 *W*_*D*_ in length, 5.4 *W*_*D*_ in height, and 5.4 *W*_*D*_ in width (Fig. 2a,b). A hexahedral volume mesh was created using the snappyHexMesh of OPENFOAM. The fluid region was divided into two parts: the overset region and the background region (Fig. 2a,b). The overset region moves and transforms to match the motion of the ray and was made with fine meshes around the analysis target and coarse meshes in the outlying areas; a one-layer boundary layer mesh was created around the analysis target. The overset region shape is an ellipsoid (Fig. 2a,b). The minimum mesh volume is 7.3 × 10^–10^ (m^3^), and the maximum mesh volume is 2.6 × 10^–2^ (m^3^). The total number of meshes was 9.0 × 10^5^. At the outlet boundary, the average static relative pressure was set to 0 Pa. The surfaces of the fish model were formed into non-slip surfaces.

The drag coefficient ${C}_{D}\left(t\right)$, the lateral force coefficient ${C}_{l}\left(t\right)$, the lift coefficient ${C}_{L}\left(t\right)$, the moment coefficient around the *x*-axis ${C}_{mx}\left(t\right)$, the moment coefficient around the *y*-axis ${C}_{my}\left(t\right)$ and the moment coefficient around the *z*-axis ${C}_{mz}\left(t\right)$ were calculated as:

9 $$\begin{array}{c}{C}_{D}\left(t\right)=\frac{D\left(t\right)}{\frac{1}{2}\rho {U}^{2}{L}_{D}{W}_{D}}\end{array}$$

10 $$\begin{array}{c}{C}_{l}\left(t\right)=\frac{l\left(t\right)}{\frac{1}{2}\rho {U}^{2}{L}_{D}{W}_{D}}\end{array}$$

11 $$\begin{array}{c}{C}_{L}\left(t\right)=\frac{L\left(t\right)}{\frac{1}{2}\rho {U}^{2}{L}_{D}{W}_{D}}\end{array}$$

12 $$\begin{array}{c}{C}_{mx}\left(t\right)=\frac{{M}_{\psi }\left(t\right)}{\frac{1}{2}\rho {U}^{2}{L}_{D}^{2}{W}_{D}}\end{array}$$

13 $$\begin{array}{c}{C}_{my}\left(t\right)=\frac{{M}_{\phi }\left(t\right)}{\frac{1}{2}\rho {U}^{2}{L}_{D}^{2}{W}_{D}}\end{array}$$

14 $$\begin{array}{c}{c}_{mz}\left(t\right)=\frac{{M}_{\theta }\left(t\right)}{\frac{1}{2}\rho {U}^{2}{L}_{D}^{2}{W}_{D}},\end{array}$$

where $D\left(t\right)$ is the calculated drag, $l\left(t\right)$ is the calculated lateral force, $L\left(t\right)$ is the calculated lift, ${M}_{\psi }\left(t\right)$ is the calculated moment around the *x*-axis, ${M}_{\phi }\left(t\right)$ is the calculated moment around the *y*-axis, ${M}_{\theta }\left(t\right)$ is the calculated moment around the *z*-axis, and $\rho$ (kg/m^3^) is the density of water at 20 °C (998 kg/m^3^). As shown in a previous study^41^. the propulsive efficiency $\eta$ is defined as the ratio of output power ${P}_{o}$ to input power ${P}_{e}$ which can be written as:

15 $$\begin{array}{c}{P}_{o}\left(t\right)=\frac{1}{T}{\int }_{0}^{T}D\left(t\right)Udt\end{array}$$

16 $$\begin{array}{c}{P}_{e}\left(t\right)=\frac{1}{T}{\int }_{0}^{T}\left[D\left(t\right)\dot{x}\left(t\right)+l\left(t\right)\dot{y}\left(t\right)+L\left(t\right)\dot{z}\left(t\right)\right]dt\end{array}$$

17 $$\begin{array}{c}\eta =\frac{{P}_{o}}{{P}_{e}}.\end{array}$$

An in-house program calculated the forces acting on rays in each axial direction and the moment around each axis*.* The numerical method's validity and reliability were verified by comparing previous experimental and numerical analytical studies of heaving and pitching on airfoil naca0013^41^. A high degree of similarity to previous studies was confirmed; the mean difference in the propulsive efficiency from the previous study of analysis was 5%, and the difference from the previous study of the experiment was 9%. Detailed information such as mesh, length, and velocity, of this analysis method's verification is provided in the “Supplement”.

A grid sensitivity study was conducted using three meshes: coarse, fine, and dense. The coarse mesh has 8.1 × 10^5^ elements, the fine mesh has 9.0 × 10^5^ elements, and the dense mesh has 9.9 × 10^5^ elements. The analysis was conducted using a condition with no phase difference of *D. matsubarai*. As shown in Fig. 2c,d, the drag coefficient and the moment coefficient around the *y*-axis are almost the same when the mesh is fine and when the mesh is dense. The mean drag and propulsive efficiency error of fine and coarse meshes are 2.7% and 3.5%, respectively. The fine mesh was used in all simulation cases considering accuracy. We used the same meshes for all cases.

## Results

### Forces acting on the body surface

**Figure 3:**
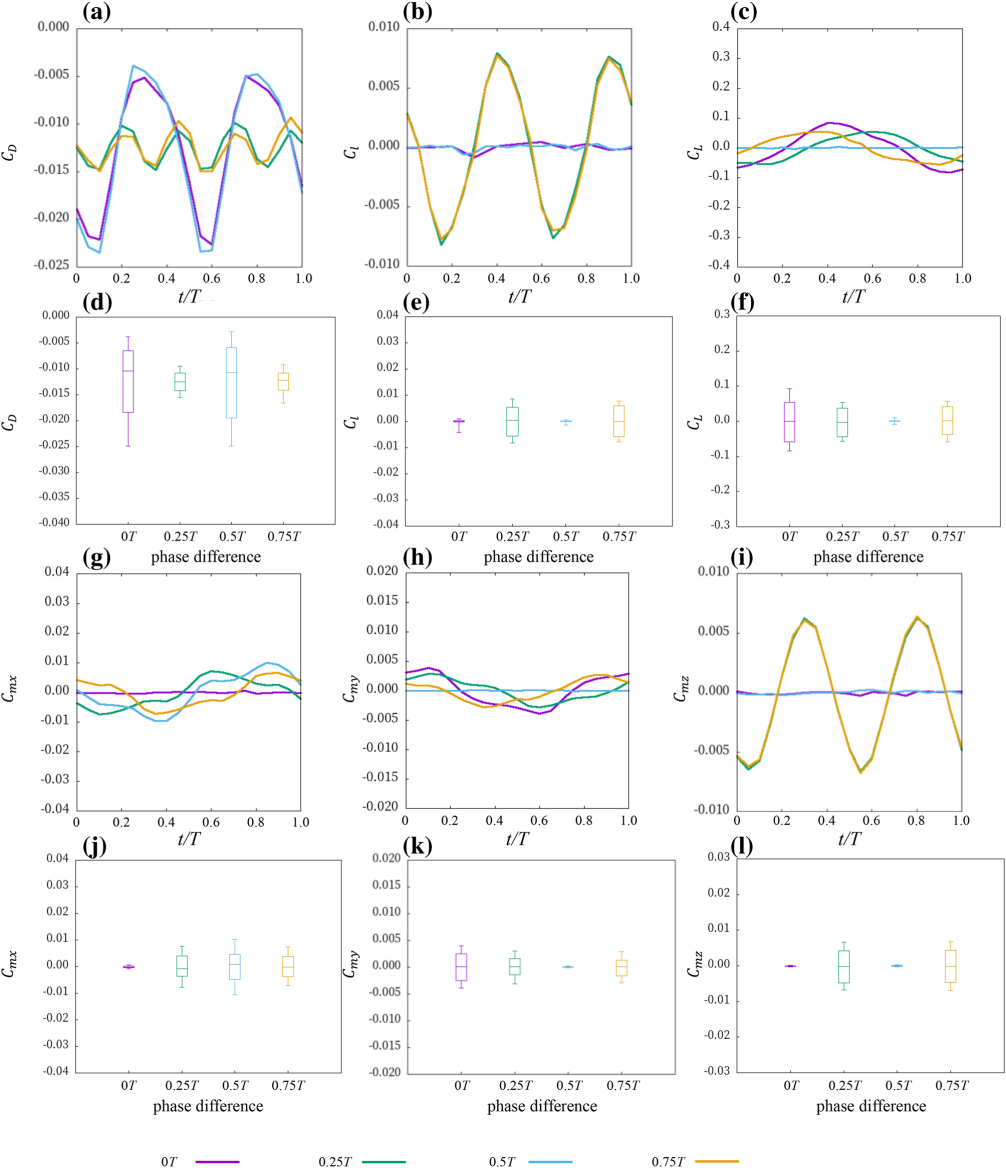
Instantaneous forces during one pectoral fin beat cycle of *D. acutirostra* forward swimming and Box-plot graph. (**a**,**d**) Drag coefficient, (**b**,**e**) lateral force coefficient, (**c**,**f)** lift coefficient, (**g**,**j**) moment coefficient around the *x*-axis, (**h**,**k**) moment coefficient around the *y*-axis, and (**i**,**l**) moment coefficient around the *z*-axis.

**Figure 4:**
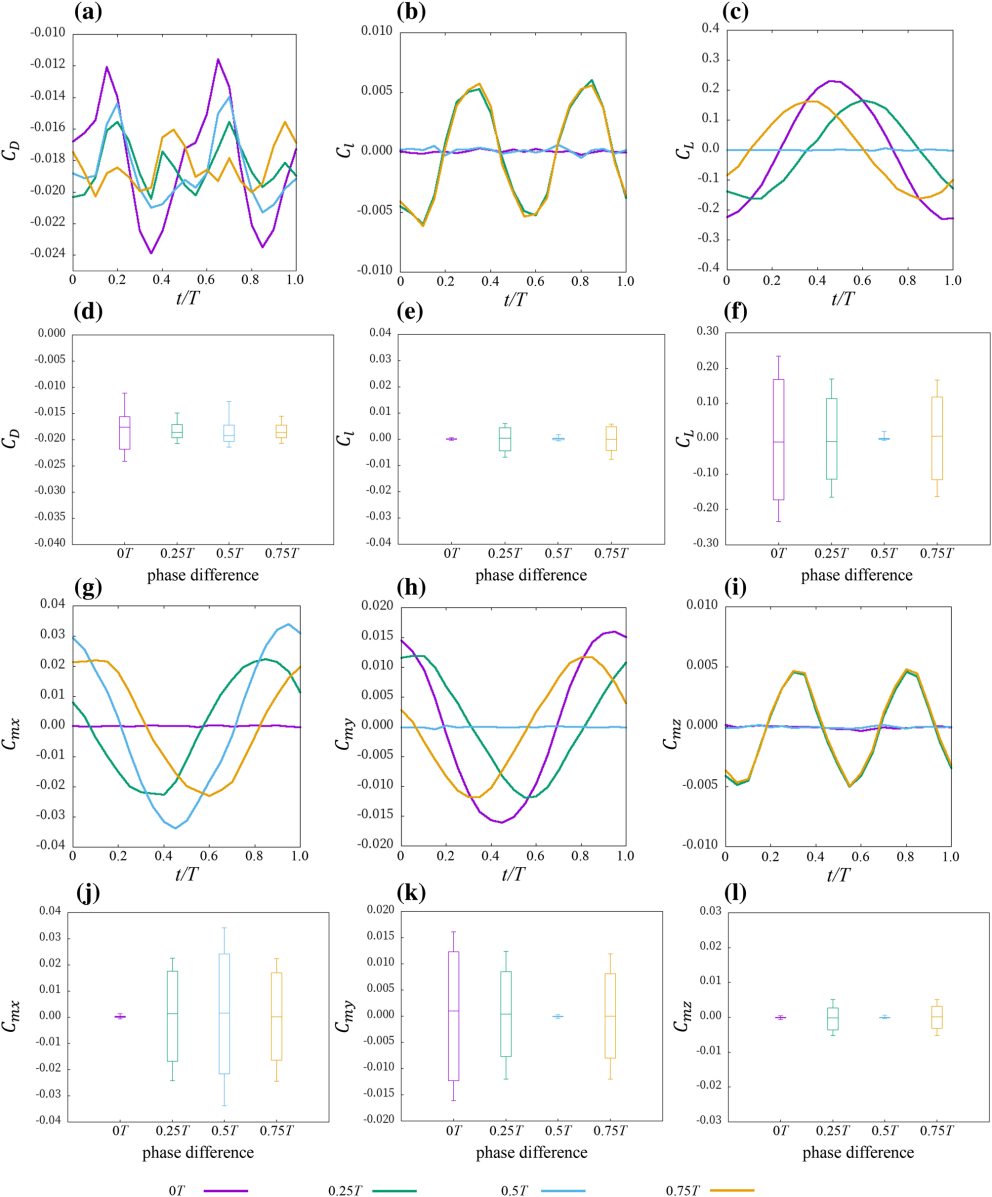
Instantaneous forces during one pectoral fin beat cycle of *D. matsubarai* forward swimming and Box-plot graph. (**a**,**d**) Drag coefficient, (**b**,**e**) lateral force coefficient, (**c**,**f**) lift coefficient, (**g**,**j**) moment coefficient around the *x*-axis, (**h**,**k**) moment coefficient around the *y*-axis, and (**i**,**l**) moment coefficient around the *z*-axis.

To investigate the effect of the phase difference of the pectoral fin motion on the force acting on the ray, we conducted an analysis in which the phase difference of the pectoral fin motion was modified. Figures 3 and 4 are the forces in each axial direction and around each axis acting on the surface of *D. acutirostra* and *D. matsubarai*, respectively. In this paper, the negative *x*-axis direction is the direction of travel, so a negative drag means that thrust is being generated.

When the phase differences of the pectoral fin shape of *D.acutirostra* were 0.25 $T$ and 0.75 $T$*,* the drag coefficient was smaller in amplitude and had twice as many peaks as in the other phase difference conditions (Fig. 3a,d). On the other hand, the pectoral fin shape of *D. matsubarai* was not as different as that of *D. acutirostra,* and the amplitude of 0 $T$ difference was slightly larger than that of the other three (Fig. 4a,d). The amplitude of the lateral force coefficient was larger in the 0.25 $T$ difference and 0.75 $T$ difference than in the other phase difference conditions in both pectoral fin shapes (Figs. 3b,e, 4b,e). Besides, it was almost zero in the case of no phase difference and 0.5 $T$ difference throughout one cycle. The lift coefficient is approximately zero only at 0.5 $T$ difference for both pectoral fin shapes throughout one cycle (Figs. 3c,f, 4c,f). In other phase difference conditions, the amplitude was changed with time, and the amplitude and period also varied with the phase difference and pectoral fin shape. The Moment coefficient around the *x*-axis is smaller in amplitude for 0 $T$ difference than for the other three in both pectoral fin shapes (Figs. 3g,j, 4g,j). As for the moment coefficient around the *y*-axis, the amplitude of 0.5 $T$ difference conditions is smaller than the other phase difference conditions (Figs. 3h,k, 4h,k). The moment coefficients around the *z*-axis, 0 $T$, and 0.5 $T$ difference were approximately zero throughout the cycle for both pectoral fin shapes. In contrast, 0.25 $T$ and 0.75 $T$ differences changed with time (Figs. 3i,l, 4i,l).

In summary, the amplitudes of 0 $T$ and 0.5 $T$ differences tended to be smaller in lateral force and the moment around the *z*-axis for both pectoral fin shapes (Figs. 3, 4b,e,i,l). The amplitude was approximately zero throughout the cycle for the lateral force, lift, moment around the *y*-axis, and the moment around the *z*-axis only under the 0.5 $T$ difference condition (Figs. 3, 4b,c,e,f,h,i,k,l). The amplitude was approximately zero throughout the cycle for the moment around the *x*-axis under the 0 $T$ difference condition (Figs. 3, 4g,j). The drag tended to vary with the pectoral fin shape (Figs. 3, 4a,d).

### Wake structure

**Figure 5:**
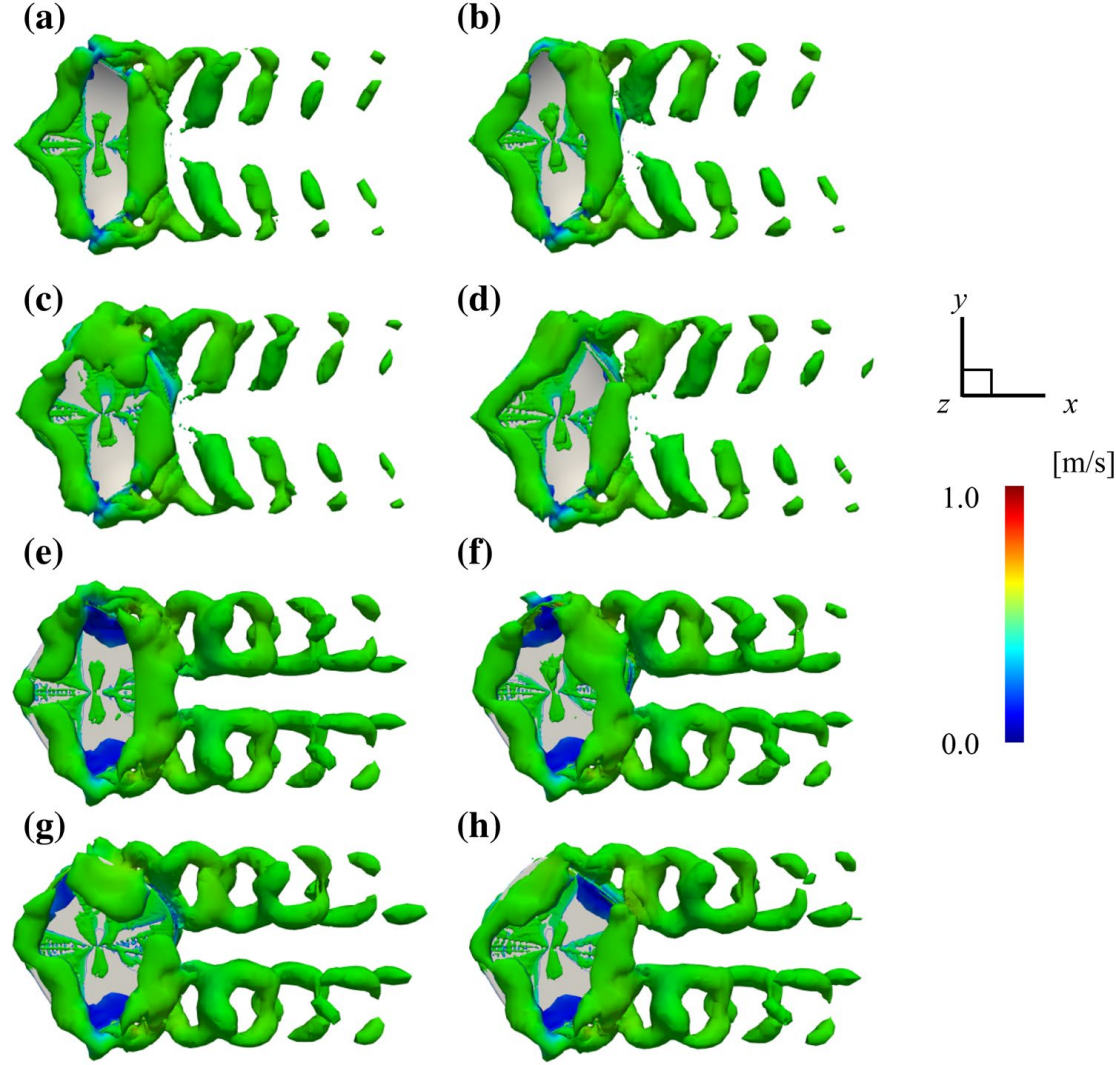
Three-dimensional wake structure of *D. acutirostra* on (**a**) 0 $T$ difference, (**b**) 0.25 $T$ difference, (**c**) 0.5 $T$ difference, and (**d**) 0.75 $T$ difference at t = 4.98. Three-dimensional wake structure of *D. matsubarai* on (**e**) 0 $T$ difference, (**f**) 0.25 $T$ difference, (**g**) 0.5 $T$ difference, and (**h**) 0.75 $T$ difference at t = 4.98.

The instantaneous three-dimensional wake structure was visualized using iso-surfaces of Q-criterion^42^ and flooded by *U*. Two rows of ring-shaped vortices are formed behind the ray (Fig. 5). These characteristics are similar to those seen in the previous study^6^. Due to the change in the phase difference of pectoral fin movements, the vortices formed posteriorly are also asymmetric. Besides, vortices generated by the left and right pectoral fins do not influence each other.

### Propulsive efficiency

The effect of the phase difference in the movement of the left and right pectoral fins on the propulsive efficiency was investigated. The results are shown in Table 1. The propulsive efficiency differed depending on the pectoral fin shape. However, there was no difference in propulsion efficiency due to the phase difference in either pectoral fin shape. The value of propulsive efficiency is more significant than that of the previous study conducted by Bottom et al.^3^ (22.87%) and close to that of the study conducted by Thekkethil et al.^6^ (approximately 38% under conditions in which the wavelength is 1.27 and the aspect ratio is 1.0).

**Table 1:** Relationship between phase difference between left and right pectoral fin movements and propulsive efficiency ($T$ is the period).

Scientific name	0T (%)	0.25T (%)	0.5T (%)	0.75T (%)
*D. acutirostra*	42.26	42.83	42.83	42.65
*D. matsubaraii*	38.57	39.20	39.75	39.23

## Discussion

We hypothesized that the phase difference in the movement of the left and right pectoral fins during straight-line swimming would increase swimming stability and maneuverability. In order to verify our hypothesis, two questions were examined: (1) What is the effect of the phase difference between the movements of the left and right pectoral fins on undulation propulsion and (2) Why do stingrays swim straight ahead by phasing their left and right pectoral fin movements. The results show that the phase difference improves swimming maneuverability and stability; our hypothesis was supported. Below are the answers to the two questions.

1. The results suggest that a phase difference of 0.5 $T$ improves swimming stability, and phase differences of 0.25 $T$ and 0.75 $T$ improve the swimming maneuverability of the rays regardless of the pectoral fin shape. In addition, propulsive efficiency was not affected by the phase differences.
2. The results of (1) suggest that rays control the phase difference to adjust swimming stability and maneuverability to suit their purpose, e.g., sudden acceleration, swimming with small oscillation, and variations in the surrounding environment^22,43^.

### Relationship between the phase difference of the pectoral fin motion and swimming performance

CFD analysis was conducted under four different phase difference conditions, and the forces acting on rays in each axial direction and the moment around each axis were compared. The results showed that the amplitude and period of each force changed with the phase difference of the left and right pectoral fin movements (Figs. 3, 4). The amplitude of the force acting on the stingray is expected to affect the oscillation of the ray's swimming motion^18^. Therefore, a small amplitude of the acting force predicts a small amount of body oscillation during swimming. Under the phase difference conditions in which we performed our experiments, the 0.5 $T$ difference is most suitable for reducing the oscillation of the swimming motion. This is because the amplitudes of all forces are smallest, except for drag and moment around the *x*-axis. On the other hand, at 0.25 $T$ and 0.75 $T$ differences, the amplitudes of most of the forces were more significant than those of the other phase difference conditions. The results suggest that the stingray's swimming will be unstable when there is a lot of oscillation, which will improve the maneuverability of the ray's swimming. Therefore, our results expected that the swimming stability of rays would be improved at 0 $T$ and 0.5 $T$ differences and that the swimming maneuverability of the ray would be improved at 0.25 $T$ and 0.75 $T$ differences. Therefore, as in previous studies^13,14,18–20^, the phase difference between the left and right pectoral fin movements may be one way to have both maneuverability and stability during straight-line swimming.

Compared to the previous study by Bottom et al.^3^, the propulsive efficiency observed is smaller. This is because the previous study considers body shape, while the current study does not. Since the body part does not generate thrust, its presence increases the projected area against the forward flow and increases the drag. In the current study, the thrust force is defined as the force acting on the rays' models in the flow direction. Therefore, the thrust and propulsive efficiency increase when the drag becomes small due to the absence of the body part. As compared with the previous study by Thekkethil et al.^6^, which did not consider the body shape, the propulsive efficiency of *D. acutirostra*, which has a pectoral fin shape similar to that of *D. matsubarai*, is almost the same.

### Mechanism by which the force acting on a ray changes due to phase differences

As shown in Fig. 5, the vortices generated by right and left pectoral fins do not interact with each other. On the other hand, if the rays have a phase difference between the left and right pectoral fin movements, the vortex structure generated posteriorly is different. For example, when there is a 0 $T$ difference, the left and right vortex structures are symmetric in the sagittal plane (Fig. 5a,e). However, under 0.5 $T$ difference conditions, the left and right vortex structures are upside down (Fig. 5c,g).

Furthermore, under 0.25 $T$ and 0.75 $T$ difference conditions, the left and right vortices are shifted in the *x*-axis direction (Fig. 5b,d,f,h). Forces acting on the stingray are generated by the vortex, while others are generated by the pectoral fins' reaction as they push against the water^5^. Thus, the forces acting on the ray consist of the above two forces generated by the left and right pectoral fins. The timing of the forces generated by the left and right pectoral fins shifted due to the phase difference between the left and right pectoral fin movements. The combined force acting on the ray was thought to have changed by the various forces strengthening or weakening each other.

### Effect of pectoral fin shape

As shown in Figs. 3 and 4, and Table 1, the phase difference between the left and right pectoral fin movements had the same effect on swimming, even though the pectoral fin shapes were different: the magnitude of the amplitude changes and the propulsive efficiency do not change. The amplitudes of the forces generated by the left and right pectoral fins were changed by the superposition of the forces generated by the left and right pectoral fins, so the effect of pectoral fin shape is small when the pectoral fins are symmetrical. Although only two pectoral fin shapes were analyzed in this study, it is expected that the phase difference between the left and right pectoral fin movements will affect the amplitude of the force acting on the ray in other pectoral fin shapes as well. However, the lift, the moment around the *x*-axis, and the moment around the *y*-axis of *D. matsubarai* are larger than those of *D. acutirostra.* It is known that swimming stability depends on the swimming motion and the shape of the body^44^. Since the motions given to the two pectoral fin shapes are the same, the shape of *D. acutirostra* might make stabilizing posture easier, and the shape of *D. matsubarai* might make stabilizing posture more difficult.

The propulsive efficiency differed depending on the pectoral fin shape. It was shown that propulsive efficiency and the force generated vary depending on the aspect ratio and the shape of the pectoral fin’s leading and trailing edges^6,29^. In this study, the models also differed in the aspect ratio and the shape of the leading and trailing edges of the pectoral fins, which resulted in differences in propulsive efficiency. In addition, the vortex structure also differed depending on the pectoral fin shape.

Vortex structures generated by *D. matsubarai'*s pectoral fin shapes similar to these of the previous study^6^ resulted in vortex structures similar to those of the previous study^6^. In contrast, vortex structures generated by *D. acutirostra'*s pectoral fin shapes different from those of the previous study^6^ resulted in vortex structures different from those of the previous study^6^. However, there are very few previous studies on different effects on swimming caused by different pectoral fin shapes. This will be clarified in our future work.

### Limitations

The motion given to the ray is not the actual motion because it was not created based on the video of the ray swimming. However, the frequency and amplitude are those of actual rays, as reported in a previous study^11^. In addition, the effect of different movements is considered to be small because the change in the force acting on the ray due to the phase difference in the direction of the pectoral fins is caused by the superposition of the forces generated by the left and right pectoral fins.

The elasticity of the pectoral fins was not taken into account in this study. However, previous studies have shown that the elasticity of the pectoral fins affects the thrust and propulsive efficiency^9,45^. Still, it is difficult to imagine that the results would differ depending on whether the pectoral fins are elastic, since the mechanism by which the phase difference affects swimming is superposition. A phase difference between the left and right pectoral fin movements is predicted to reduce swimming oscillation even when elasticity is considered. It will be investigated in our future work.

Inertial forces and fluid resistance are expected to change under the actual movement of rays. Since this study is not a self-propelled analysis, it does not consider the effects of the ever-changing behavior of rays. Therefore, in discussing the stability of rays, it is best to consider the movement of rays due to inertial and hydrodynamic forces^21^. However, the phase difference between the left and right pectoral fin movements of the ray is expected to affect the ray's stability during self-propulsion because its stability will undoubtedly change as the amplitude of the force acting on the ray changes. Therefore, in our future work, we will investigate how the phase difference between the movements of the left and right pectoral fins affects the self-propulsion of rays.

### Biological implications and biomimetics applications

Many studies on rays' swimming have been conducted, and rays' propulsion efficiency and swimming mechanism have been clarified^3–6,9,11,12,24,45^. Most previous studies have been undertaken with symmetrical swimming motions, but actual rays have a phase difference during swimming, which is important to note. Shi et al. conducted a fluid analysis of a stingray-type robot and found that the phase difference between the movements of the left and right pectoral fins improves turning ability^12^. However, the effect of phase differences in pectoral fin movements on straight-line swimming in rays has not yet been elucidated. Therefore, we conducted fluid dynamics analysis under four phase difference conditions to investigate the effect of the phase difference on left and right pectoral fin movements of rays during swimming. Our results showed that the force acting on the rays changed when the phase difference was changed. However, the propulsion efficiency was not affected by the phase difference. These results suggest that rays change the phase difference between the left and right pectoral fin movements according to their purpose. Therefore, when studying the swimming behavior of stingrays, it is better to account for the phase difference between right and left pectoral fins to obtain detailed results on swimming mechanisms and ecology.

Moreover, stingray-shaped underwater robots are being developed^12,14,24,45,46^. It is essential to reduce the underwater robot's motion oscillation when swimming for investigative work underwater. However, additional energy is often used to maintain a constant attitude^47^. This study shows that controlling posture by changing the phase difference between the left and right pectoral fins does not change the propulsive efficiency. Therefore, we propose that phase differences effectively eliminate oscillation due to swimming motion during swimming without degrading propulsive efficiency.

## Supplementary Information

Supplementary Information.

Supplementary Video 1.

Supplementary Video 2.

Supplementary Video 3.

Supplementary Video 4.

## Data Availability

We conducted CFD analysis using OPENFOAM; it is open-source so that anyone can get the numerical analysis code. Moreover, the data, the code for swimming movement, and the set of codes used for the analysis are available from the corresponding author upon request.
