# Supplementary material for: Changes in rays’ swimming stability due to the phase difference between left and right pectoral fin movements
Source: Sci Rep. 2022 Feb 11;12:2362. doi: 10.1038/s41598-022-05317-5 (PMC8837794; doi:10.1038/s41598-022-05317-5)
Supplement: Supplementary file 1 — Supplementary Information. [file 41598_2022_5317_MOESM1_ESM.pdf]

The Electronic Supplementary Materials (ESM) in the article "Changes in rays' swimming stability due to the phase difference between left and right pectoral fin movements."

The file contains:

1) The model and the methods of verification and validation,
2) Figures S1, S2,
3) Legends of the supplementary videos
4) ESM references.

The analytical target and methods of CFD analysis

To evaluate the validity of our analysis method, we compared it with a previous study conducted by Zhou et al.[s1] The model, motion, and Reynolds number used in our analysis are the same as those used in the previous study, and the details are described below.

A CFD simulation was performed using the open-source finite volume method CFD toolbox (OpenFOAM-v1806[s2]) to calculate the drag, lateral force, lift, and propulsive efficiency and compared it with the previous work[s1]. The governing equations were the continuity equation and the three-dimensional incompressible Reynolds-averaged Navier-Stokes equation, stated in the main manuscript. The Reynolds number was defined as:

$$R_e = \frac{UL}{\nu} \ , \tag{1}$$

where U(ms^{-1}) is the applied constant flow speed, L is the chord length of NACA0013, and ν is the kinematic viscosity of water at 20 ºC (1.004×10^{-6} m^{2}s^{-1}). The Reynolds number of this analysis is 1.1×10^4. Considering this, we used *k-ω* shear stress turbulence model[s3]; the OpenFOAM variant is based on the 2003 *k-ω* shear stress turbulence model[s3]. The overset grid method was used in this study; it is a generic implementation of overset meshes. For both static and dynamic cases, the cell-to-cell mapping between multiple, disconnected mesh regions is employed to generate a composite domain [s4,s5]. This method permits complex mesh motions and interactions without the penalties associated with deforming meshes. The process is described in detail in Noack [s6]. The calculation volume was 1.1 m in length, 0.5 m in height, and 0.8 m in width (Fig.S1). A hexahedral volume mesh was created using the snappyHexMesh of OpenFOAM®. The fluid region was made with fine meshes around the analysis target and coarse meshes in the outlying areas; a 5-layer boundary layer mesh was created around the analysis target. The minimum mesh volume is 1.6×10^{-11} m^3 and the maximum mesh volume is 1.1×10^{-5} m^3. The total number of meshes was 5.0×10^6 elements. A constant longitudinal flow speed with 5 % turbulence intensity was applied at the inlet boundary. At the outlet

boundary, the average static relative pressure was set to 0 Pa. The surface of the caudal fin model and surrounding walls were formed into a non-slip surface.

The motion used in the analysis is a combination of heave $y(t)$ and pitch $\theta(t)$. The equation can describe the heave motion of the fin:

$$y(t) = h\,cos(2\pi f t) \tag{2}$$

where f is the flapping frequency, t is the time, and h is the amplitude of the heave motion. In addition, the pitching angle θ is given by:

$$\theta(t) = \alpha(t) + \theta_m(t) \tag{3}$$

where

$$\theta_m(t) = arctan\left(\frac{\dot{y}(t)}{U}\right) \tag{4}$$

The angle $\alpha(t)$ is the angle of attack and given by:

$$\alpha(t) = \alpha_{max}\,sin(2\pi f t) \tag{5}$$

Where α_{max} is amplitude of the angle of attack.

As shown in the previous study[s1], the forces and torque are converted to dimensionless forms and should be described by:

$$C_x(t) = \frac{F_x(t)}{0.5\rho U^2 S} \tag{6}$$

$$C_y(t) = \frac{F_y(t)}{0.5\rho U^2 S} \tag{7}$$

$$C_m(t) = \frac{M_\theta(t)}{0.5\rho U^2 SL} \tag{8}$$

Where ρ is the density of water at 20 °C (998kgm^{-3}), F_y is the lateral force, F_x is the thrust force, M_θ is the torque, and S is the projected area of the fin (one-sided). As shown in the previous study[s1], the propulsive efficiency η is defined as the ratio of output power P_o to input power P_e which can be written as:

$$P_o(t) = \frac{1}{T}\int_0^T F_x(t) U\,dt \tag{9}$$

$$P_e(t) = \frac{1}{T}\int_0^T \left[F_y(t)\dot{y}(t) + M_z(t)\dot{\theta}(t)\right]dt \tag{10}$$

$$\eta = \frac{P_o}{P_e} \tag{11}$$

Where T is the period of the motion of the NACA0013.

The forces were calculated from friction and pressure, which were separately output by OpenFoam's sampling utility sampleDict. The average wall-normal distance of the cell center in wall units, y^+, of NACA0013, is 0.34. OpenFOAM's wall

function models of omegaWallFunction and kqRWallFunction were used as wall function models. These wall function models estimated the cell center's wall-normal distance and the intersection of the viscous and inertial sublayers in wall units. The viscous and inertial sublayer estimations of specific dissipation rates are switched between each other depending on the y^+ value of the point of intersection of the viscous and inertial sublayers in-wall units[s7,s8]. The time step during the simulation was adjusted according to the maximum courant number 0.2.

In the results, the period and maximum values of drag, lateral force, and torque were in close agreement with the previous studies[s1], as shown in Fig.S2. Moreover, the propulsive efficiency calculated by the present analytical method was 44 %, which was 5 % different from the analytical value in the previous study and 9 % different from the experimental value in the previous study. The reason for the error may be due to differences in solvers or measurement errors. However, the magnitudes and trajectories of drag, lateral force, and torque are almost identical, and the error in propulsive efficiency is one order of magnitude, so the validity of this method is sufficient.

Figures

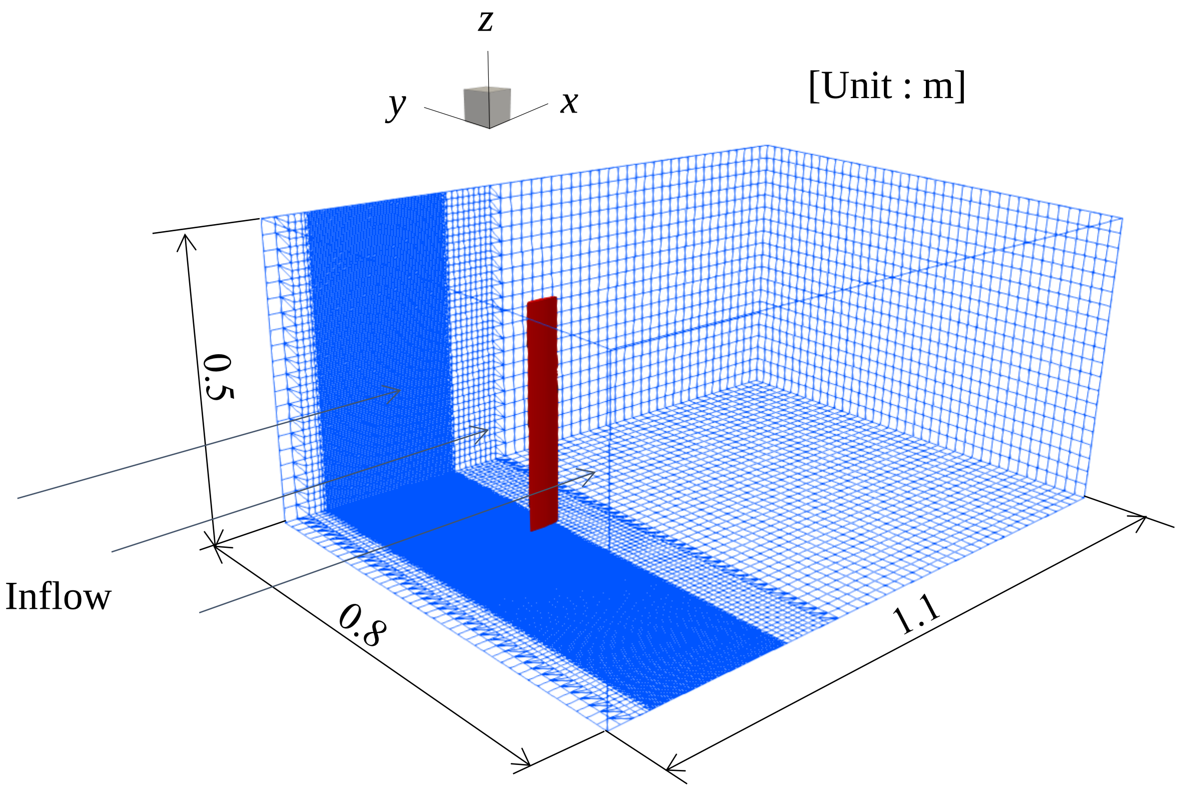

Fig.S1 Analytical region and model

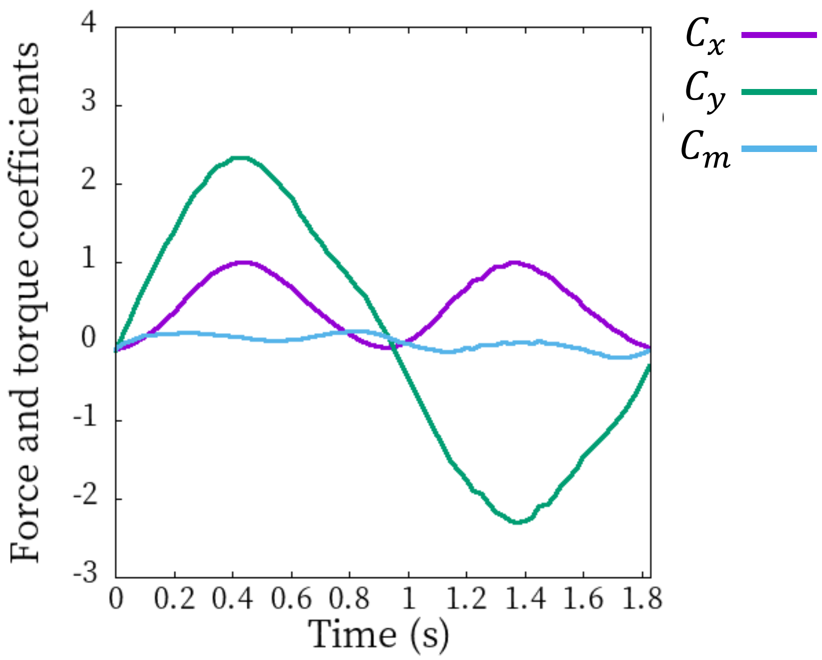

Fig. S2 Performance curves of flapping foil

Legends of the supplementary videos

Supplement movie1: *D. matsubarai* swimming at the KAIYUKAN.

Supplement movie2: *D. acutirostra* swimming at the KAIYUKAN.

Supplement movie3: The created motion of *D. matsubarai* from the front when there is no phase difference.

Supplement movie4: The created motion of *D. matsubarai* from the left side when there is no phase difference.

References

s1. Zhou, K., Liu, J. & Chen, W. Numerical study on hydrodynamic performance of bionic caudal fin. *Appl. Sci. (Basel)* **6**, 15 (2016).

s2. OpenCFD. OpenFOAM® - Official home of The Open Source Computational Fluid Dynamics (CFD) Toolbox. https://www.openfoam.com/.

s3. OpenFOAM: User Guide: k-omega Shear Stress Transport (SST). https://www.openfoam.com/documentation/guides/latest/doc/guide-turbulence-ras-k-omega-sst.html.

s4. OpenFOAM: User Guide: Overset. https://www.openfoam.com/documentation/guides/latest/doc/guide-overset.html.

s5. Shen, Z., Wan, D. & Carrica, P. M. Dynamic overset grids in OpenFOAM with application to KCS self-propulsion and maneuvering. *Ocean Eng.* **108**, 287–306 (2015).

s6. Noack, R. SUGGAR: A General Capability for Moving Body Overset Grid Assembly. in *17th AIAA Computational Fluid Dynamics Conference* (American Institute of Aeronautics and Astronautics, 2005).

s7. Menter, F., Kuntz, M. & Langtry, R. Ten Years of Industrial Experience with the SST Turbulence Model. (2003).

s8. OpenFOAM: User Guide: omegaWallFunction. https://www.openfoam.com/documentation/guides/latest/doc/guide-bcs-wall-turbulence-omegaWallFunction.html.
